# Roles of Macrophage Exosomes in Immune Response to Calcium Oxalate Monohydrate Crystals

**DOI:** 10.3389/fimmu.2018.00316

**Published:** 2018-02-27

**Authors:** Nilubon Singhto, Rattiyaporn Kanlaya, Angkhana Nilnumkhum, Visith Thongboonkerd

**Affiliations:** ^1^Medical Proteomics Unit, Office for Research and Development, Faculty of Medicine Siriraj Hospital, Mahidol University, Bangkok, Thailand; ^2^Immunology Graduate Program, Department of Immunology, Faculty of Medicine Siriraj Hospital, Mahidol University, Bangkok, Thailand; ^3^Center for Research in Complex Systems Science, Mahidol University, Bangkok, Thailand

**Keywords:** calcium oxalate, calcium oxalate monohydrate, inflammasome, inflammation, kidney stone, migration, phagocytosis

## Abstract

In kidney stone disease, macrophages secrete various mediators *via* classical secretory pathway and cause renal interstitial inflammation. However, whether their extracellular vesicles, particularly exosomes, are involved in kidney stone pathogenesis remained unknown. This study investigated alterations in exosomal proteome of U937-derived macrophages (by phorbol-12-myristate-13-acetate activation) after exposure to calcium oxalate monohydrate (COM) crystals for 16-h using 2-DE-based proteomics approach. Six significantly altered proteins in COM-treated exosomes were successfully identified by nanoscale liquid chromatography–electrospray ionization–electron transfer dissociation tandem mass spectrometry as proteins involved mainly in immune processes, including T-cell activation and homeostasis, Fcγ receptor-mediated phagocytosis, interferon-γ (IFN-γ) regulation, and cell migration/movement. The decreased heat shock protein 90-beta (HSP90β) and increased vimentin were confirmed by Western blotting. ELISA showed that the COM-treated macrophages produced greater level of interleukin-1β (IL-1β), one of the markers for inflammasome activation. Functional studies demonstrated that COM-treated exosomes enhanced monocyte and T-cell migration, monocyte activation and macrophage phagocytic activity, but on the other hand, reduced T-cell activation. In addition, COM-treated exosomes enhanced production of proinflammatory cytokine IL-8 by monocytes that could be restored to its basal level by small-interfering RNA targeting on vimentin (si-Vimentin). Moreover, si-Vimentin could also abolish effects of COM-treated exosomes on monocyte and T-cell migration as well as macrophage phagocytic activity. These findings provided some implications to the immune response during kidney stone pathogenesis *via* exosomal pathway of macrophages after exposure to COM crystals.

## Introduction

During an initial phase of kidney stone formation, the causative chemical crystals, such as calcium oxalate (CaOx), can be deposited in the renal interstitium, where macrophages are recruited to eliminate these crystals *via* phagocytosis (1–3). Between the two common hydrated forms of CaOx crystals, calcium oxalate monohydrate (COM) crystals are predominantly found in clinical stones, whereas CaOx dihydrate (COD) crystals can be also found but with smaller proportion (4). Due to differences in adhesive capability, binding kinetics, atomic lattice, and surface ionic pattern, COM crystals are more pathogenic during the kidney stone pathogenesis than COD crystals, which can be also found in the normal urine of healthy individuals (5–9).

Several lines of evidence have shown that macrophages exposed to COM crystals increase secretion of reactive oxygen species (ROS), chemokines, proinflammatory cytokines, and several fibrotic factors to promote renal interstitial inflammation in kidney stone disease (10–12). The COM-phagocytosed macrophages can activate NACHT, leucine-rich repeat (LRR), and pyrin domain-containing protein 3 (NLRP3), which is the central molecule triggering vascular permeability, leukocyte recruitment, complement activation, and inflammatory mediator production (13, 14). NLRP3-inflammasome-activated macrophages can secrete several proinflammatory cytokines, including interleukin-1β (IL-1β), IL-6, and IL-18, which serve as the amplification loop factors to activate tubulointerstitial damage by stimulating the recruited inflammatory cells (15, 16). Additionally, macrophages exposed to naturally occurred kidney stone fragments secrete greater levels of several chemokines, particularly macrophage inhibitory protein-1, monocyte chemoattractant protein-1, and interleukin-8 (IL-8) (17). These chemokines consequently enhance recruitment of various immune cells, i.e., monocytes, macrophages, neutrophils, dendritic cells, and T-cells into the inflammatory locale (18).

In addition to these inflammatory/proinflammatory mediators, macrophages can also secrete nanovesicles with a discrete diameter of approximately 30–100 nm, namely “exosomes,” which play pivotal roles in intercellular communications and multibiological functions (19). Nevertheless, whether exposure to COM crystals causes any alterations in macrophage exosomes remained unknown. This study thus aimed to investigate alterations in exosomal proteins after macrophages were exposed to COM crystals using a proteomics approach followed by validation of expression data as well as several functional assays to address functional significance of exosomes derived from COM-treated macrophages in relation to kidney stone pathogenesis, particularly during an induction phase of renal interstitial inflammation.

## Materials and Methods

### COM Crystal Preparation

Calcium oxalate monohydrate crystals were prepared as described previously (20, 21). Briefly, 10 mM CaCl_2_.2H_2_O was mixed with 1.0 mM Na_2_C_2_O_4_ (1:1 v/v) to make their final concentrations to 5 and 0.5 mM, respectively, in a buffer containing 10 mM Tris-HCl and 90 mM NaCl (pH 7.4). After incubation at 25°C overnight, COM crystals were harvested by a centrifugation at 2,000 *g* for 5 min. The supernatant was discarded, whereas COM crystals were washed three times with methanol. After another centrifugation at 2,000 *g* for 5 min, methanol was discarded and the crystals were air-dried overnight at 25°C. The typical morphology of COM crystals was examined under an inverted phase-contrast light microscope (model ECLIPSE Ti-S, Nikon; Tokyo, Japan).

### Cell Culture and Macrophage Differentiation

U937 human monocytic cell line and Jurkat T-cell line were cultivated and maintained in complete RPMI 1640 medium (Gibco; Grand Island, NY, USA) supplemented with 10% (v/v) heat-inactivated fetal bovine serum (FBS) (Gibco), 100 U/ml penicillin G and 100 mg/ml streptomycin (Sigma, St. Louis, MO, USA).

Macrophages were derived from U937 human monocytic cell line using phorbol 12-myristate 13-acetate (PMA) (Fluka, St. Louis, MO, USA) for differentiation as previously described (22). Briefly, U937 monocytic cells at a density of 1 × 10^6^ cells/ml were treated with 100 ng/ml PMA for 48 h (induction phase) and then vigorously washed three times with ice-cold PBS to remove PMA and non-adherent cells, whereas the adherent cells were further maintained as aforementioned for 48 h (recovery phase). The characteristics of macrophages were observed under an inverted phase-contrast microscope (Nikon ECLIPSE Ti-S) as previously described (22).

### COM Crystal Treatment

The COM crystals were decontaminated by exposure to UV light for 30 min prior to incubation with the cells. After recovery phase, U937-derived macrophages (10 × 10^6^ cells/flask) were vigorously washed five times with ice-cold PBS to remove serum-containing medium and further cultivated in serum-free medium with or without 100 µg/ml COM crystals for 16 h, which was the optimal time-point defined for studying macrophage secretome as previously reported (22) (*n* = 5 independent culture flasks per group; a total of 10 independent cultures were subjected to 2-DE analysis, whereas three independent biological replicates were used for other experiments). After 16-h incubation, the culture supernatants were harvested and further subjected to exosome isolation as detailed below.

### Exosome Isolation by Microfiltration and Differential Centrifugation

The controlled and COM-treated macrophage supernatants were filtrated through 0.22-µm cellulose acetate membrane (Sartorius Stedim Biotech GmbH, Goettingen, Germany) to remove cell debris and apoptotic bodies. Microvesicles and/or larger vesicles were further removed by centrifugation at 10,000 *g* and 25°C for 30 min. Exosomes were then isolated from the remaining supernatants by ultracentrifugation at 100,000 *g* and 25°C for 90 min using an ultracentrifuge (Sorvall, Langenselbold, Germany). The isolated exosomal pellets were washed twice with PBS and resuspended in 2% (w/v) paraformaldehyde or a lysis buffer (based on experiments described below).

### Examination of Exosome Morphology by Transmission Electron Microscopy (TEM)

Exosomes were resuspended in 2% (w/v) paraformaldehyde and loaded onto carbon-Formvar-coated copper grids. The samples were left on the grids for 20 min to adsorb and form monolayers. The remaining samples were washed three times with PBS. The grids were then fixed with 50 µl of 2% (v/v) glutaraldehyde for 5 min and subsequently washed eight times with distilled water. The grids were contrasted with 50 µl of 4% (v/v) uranyl acetate (pH 7.0) for 5 min and the excess fluid was then removed by filter paper. Finally, the grids were loaded onto a transmission electron microscope (Tecnai G2 TEM Series, Hillsboro, OR, USA) with an accelerating voltage set at 80 kV with a magnification of 250,000×.

### Exosomal Protein Extraction

Exosomes were resuspended in a 2-D lysis buffer containing 7 M urea, 2 M thiourea, 4% 3-[(3-cholamidopropyl)-dimethyl-ammonio]-1-propanesulfonate (CHAPS), 120 mM dithiothreitol (DTT), 2% ampholytes (pH 3−10), and 40 mM Tris-HCl and incubated at 4°C for 30 min. Protein concentrations were measured by the Bradford’s method using Bio-Rad protein assay (Bio-Rad, Milano, Italy).

### Western Blotting

Equal amount of exosomal proteins (20 μg/sample) from each sample were mixed with 2× Laemmli’s buffer (to make the final concentration of 1× Laemmli’s buffer) and resolved by 12% SDS-PAGE at 150 V for approximately 2 h using SE260 mini-Vertical electrophoresis unit (GE Healthcare; Uppsala, Sweden). After the completion of SDS-PAGE, the resolved proteins were transferred onto a nitrocellulose membrane (Whatman, Dassel, Germany) using a semidry transfer apparatus (GE Healthcare) at 85 mA for 1.5 h. Non-specific bindings were blocked with 5% skim milk in PBS at 25°C for 1 h. The membrane was incubated with mouse monoclonal anti-heat shock protein 70 (anti-HSP70), anti-Rab5, anti-HSP90β, anti-vimentin, or rabbit polyclonal anti-Rab7 antibody (all were purchased from Santa Cruz Biotechnology, Santa Cruz, CA, USA and were diluted 1:1,000 in 1% skim milk/PBS) at 4°C overnight. After washing with PBS three times, the membrane was incubated with corresponding secondary antibody conjugated with horseradish peroxidase (1:2,000 in 1% skim milk/PBS; DAKO Glostrup, Denmark) at 25°C for 1 h. Immunoreactive bands were developed by SuperSignal West Pico chemiluminescence substrate (Pierce Biotechnology, Rockford, IL, USA) and were then visualized by autoradiogram.

### 2-DE and Staining

Exosomal proteins derived from each culture flask were resolved in each 2-D gel as previously described (21, 23) (60 µg total protein/each sample/gel; *n* = 5 gels/group; a total of 10 gels were analyzed). Each protein sample was premixed with a rehydration buffer containing 7 M urea, 2 M thiourea, 2% CHAPS, 120 mM DTT, 40 mM Tris-base, 2% ampholytes (pH 3–10), and a trace of bromophenol blue to make a final volume of 150 µl. The mixture was rehydrated onto an Immobiline DryStrip (nonlinear pH gradient of 3–10, 7 cm long) (GE Healthcare, Uppsala, Sweden) at 25°C for 10–15 h. The first dimensional separation or isoelectric focusing (IEF) was performed in Ettan IPGphor III IEF System (GE Healthcare) at 20°C, using a stepwise mode to reach 9,083 Vh with a limiting current of 50 mA/strip. The IPG strips were then incubated for 15 min in equilibration buffer I containing 6 M urea, 130 mM DTT, 112 mM Tris-base, 4% SDS, 30% glycerol, and 0.002% bromophenol blue following by another 15 min in equilibration buffer II containing similar compositions as of buffer I, but DTT was replaced with 135 mM iodoacetamide. The equilibrated IPG strips were subjected to the second dimensional separation in 12.5% SDS-polyacrylamide gel using SE260 Mini-Vertical Electrophoresis Unit (GE Healthcare) at 20 μA/gel for approximately 1.5 h. Thereafter, the resolved proteins were stained with Deep Purple protein fluorescence dye (GE Healthcare) and visualized by using Typhoon 9200 laser scanner (GE Healthcare).

### Spot Matching and Quantitative Intensity Analysis

Protein spots visualized in 2-DE gels were analyzed using ImageMaster 2D Platinum software (GE Healthcare). Parameters used for spot detection were (i) minimal area = 10 pixels; smooth factor = 2.0 and (ii) saliency = 200. A reference gel was created from an actual gel with the greatest number of protein spots and additional spots that were present in other gels were also combined to produce a single artificial reference gel with all protein spots present in all gels. The reference gel was then used for matching the corresponding protein spots across different gels. Background subtraction was performed and the intensity volume of each spot was normalized with total intensity volume (summation of the intensity volumes obtained from all spots within the same 2-D gel). Differentially expressed protein spots that reached statistically significant threshold (*P* < 0.05) were subjected to in-gel tryptic digestion and identification by mass spectrometry.

### In-gel Tryptic Digestion

In-gel tryptic digestion was performed following protocol described previously (24, 25). Briefly, the protein spots with significantly differential levels were excised from 2-D gels, washed with 1 ml deionized water, and then destained with 100 µl of 100 mM NH_4_HCO_3_ at 25°C for 15 min. Thereafter, 100 µl acetonitrile (ACN) was added and incubated at 25°C for 15 min. After removing the solvent, the gel pieces were dried in a SpeedVac concentrator (Savant; Holbrook, NY, USA) and rehydrated with 50 µl of 10 mM DTT in 100 mM NH_4_HCO_3_ at 56°C for 30 min using a heat box. After removing the reducing buffer, the gel pieces were incubated with 50 µl of 55 mM iodoacetamide in 100 mM NH_4_HCO_3_ at 25°C for 20 min in the dark. The buffer was then removed, whereas the gel pieces were incubated with 100 µl of 50 mM NH_4_HCO_3_ at 25°C for 15 min. Thereafter, 100 µl ACN was added and incubated at 25°C for 15 min. After removing the solvent, the gel pieces were dried in a SpeedVac concentrator, and then incubated with a minimal volume (just to cover gel pieces) of 12 ng/µl sequencing grade modified trypsin (Promega, Madison, WI, USA) in 50 mM NH_4_HCO_3_ in a ThermoMixer^®^ C (Eppendorf, Hauppauge, NY, USA) at 37°C for 16–18 h. The digestion reaction was stopped by incubation with 100 µl of 5% formic acid/ACN (1:2 vol/vol) at 37°C for 15 min. The digested peptide mixtures were collected using a pipette with gel loader tip, transferred into a fresh tube, dried by a SpeedVac concentrator, and subjected to MS/MS analysis.

### Identification of Proteins by Nanoscale Liquid Chromatography–Electrospray Ionization–Electron Transfer Dissociation Tandem Mass Spectrometry (nanoLC-ESI-ETD MS/MS)

Separation of the digested peptides was performed using EASY-nLC II (Bruker Daltonics, Bremen, Germany) as previously described (26, 27). Briefly, peptides were loaded from a cooled (7°C) autosampler into an in-house, 3-cm-long pre-column containing 5-µm C18 resin (Dr. Maisch GmbH, Ammerbuch, Germany) and then to an in-house, 10-cm-long analytical column packed with 3-µm C18 resin (Dr. Maisch GmbH) using mobile phase A (0.1% formic acid). The peptides were then separated by mobile phase B (ACN/0.1% formic acid) gradient elution with three steps as follows: 0–35% for 30 min, 35–80% for 10 min, and then 80% for 10 min at a flow rate of 300 nl/min. Peptide sequences were then analyzed by amaZon speed ETD (Bruker Daltonics) with ESI nanosprayer ion source (spray capillary: fused silica with outer diameter of 90 µm and inner diameter of 20 µm) controlled by HyStar version 3.2 and trapControl version 7.1. Mass spectrometric parameters were set as follows: electrospray voltage = 4,500 V, high-voltage end-plate offset = 500 V, nebulizer gas = 0.55 bar, dry gas = 5.0 l/min, and dry temperature = 150°C. Precursors were scanned from 400 to 2,200 *m/z* range with enhanced resolution mode (speed = 8,100 *m/z*/s), ion charge control (ICC) target = 200,000, maximal accumulation time = 50 ms. The three most intense signals in every MS scan were selected for MS/MS analysis, whereas singly charged ions were excluded. For MS/MS experiment, fragmented peptides from 150 to 3,000 *m/z* range were scanned with XtremeScan mode (speed = 52,000 *m/z*/sec), ICC target = 200,000, maximal accumulation time = 100 ms. Mass spectra were deconvoluted *via* DataAnalysis version 4.0 SP5 (BrukerDaltonics) to .*mgf* file. Mascot software version 2.4.0 (Matrix Science; London, UK) was used to search MS/MS spectra against NCBI database of mammalian with the following standard Mascot parameters for CID: Enzyme = trypsin, maximal number of missed cleavages = 1, peptide tolerance = ± 1.2 Da, MS/MS tolerance = ± 0.6 Da, fixed modification = carbamidomethyl (C), variable modification = oxidation (M), charge states = 2+ and 3+, and instrument type = ESI-Trap.

### Effect of COM Crystals on Inflammasome Activation

To evaluate the effect of COM crystal treatment on inflammasome activation, the culture supernatants derived from the controlled and COM-treated macrophages (2 × 10^6^ cells/well) were collected, clarified by centrifugation at 300 *g*, and then subjected to indirect ELISA to measure level of IL-1β, one of the markers of inflammasome activation. Briefly, the clarified culture supernatant was concentrated by vacuum concentrator until completely dried. Thereafter, the samples were resuspended in 50 µl coating buffer (15 mM Na_2_CO_3_ and 30 mM NaHCO_3_; pH 9.4) and then coated onto 96-well ELISA plate (Nunc, Roskilde, Denmark) at 4°C overnight. After washing with a washing buffer [0.05% (v/v) Tween-20/PBS], non-specific bindings were blocked by 1%BSA/PBS at 25°C for 2 h. After another wash, 100 µl of hamster monoclonal anti-IL-1β primary antibody (Santa Cruz Biotechnology) (diluted 1:50 in 0.1% BSA/PBS) was added and incubated at 25°C for 2 h. After another wash, the corresponding secondary antibody conjugated with horseradish peroxidase (diluted 1:100 in 0.1% BSA/PBS) was added and further incubated at 25°C for 2 h in the dark. The plate was then washed and added with 100 µl substrate solution (1.5 mM ortho-phenylenediamine dihydrochloride in 35 mM citric acid and 0.012% H_2_O_2_; pH 5.5). The reaction was allowed for 15 min in the dark before 50 µl of stop reaction solution (2 M H_2_SO_4_) was added. Finally, the absorbance (optical density) of the sample was measured at λ492 nm using an ELISA plate reader (Biochrom Ltd., Cambridge, UK).

### Effects of COM-Treated vs. Controlled Exosomes on Monocyte and T-Cell Migration

Effects of exosomes derived from untreated (controlled exosomes) vs. COM-treated macrophages (COM-treated exosomes) on the migratory ability of monocytes and T-cells were evaluated using transwell culture plates with 5-µm pore size (Corning Life Sciences; Tewksbury, MA, USA) following protocol described previously with slight modification (3). Briefly, a total of 2 × 10^5^ cells/well of U937 monocytes and Jurkat T-cells were cocultivated with 30 µg intact controlled or COM-treated exosomes at the upper chamber of transwell containing serum-free medium. To provide chemoattractant gradient, the medium at the lower chamber was supplemented with 10% FBS. After 24-h incubation, numbers of monocytes and T-cells migrated from upper to lower chambers were observed under an inverted phase-contrast light microscope (Nikon ECLIPSE Ti-S) and counted from at least 10 low-power fields (LPF) using ImageJ software (version 1.50f) (http://imagej.nih.gov/ij).

### Effects of COM-Treated vs. Controlled Exosomes on Monocyte and T-Cell Activation

To evaluate effects of exosomes derived from untreated (controlled exosomes) vs. COM-treated macrophages (COM-treated exosomes) on monocyte and T-cell activation, flow cytometric analyses of markers for activated monocytes (CD11b) and T-cells (CD69) were performed. Following the migration assay as described above, the migrated cells at lower chamber of transwell were fixed with 2% (w/v) paraformaldehyde at 25°C for 15 min. Non-specific bindings were blocked with 5% (w/v) BSA in PBS and the cells were incubated with 1 µg/10^6^ cells mouse monoclonal anti-CD11b or anti-CD69 antibody (both were from Santa Cruz biotechnology and were diluted in 1% BSA/PBS) at 25°C for 1 h. Thereafter, the cells were incubated with rabbit anti-mouse IgG conjugated with Alexa 488 (Molecular probe, Invitrogen; Eugene, OR, USA) (1:2,000 in 1%BSA/PBS) at 25°C for 1 h. The cells were then washed twice with ice-cold PBS and further analyzed by a flow cytometer (FACaliburs, Becton Dickinson Immunocytometry System, San Jose, CA, USA). IgG1 isotype antibody was used as the negative control.

### Effects of COM-Treated vs. Controlled Exosomes on Macrophage Phagocytic Activity

To evaluate effects of exosomes derived from untreated (controlled exosomes) vs. COM-treated macrophages (COM-treated exosomes) on phagocytic activity, macrophages (2 × 10^5^ cells/well) were incubated with 30 µg of intact controlled or COM-treated exosomes for 24 h. Thereafter, approximately 2 × 10^7^ cells of *Saccharomyces cerevisiae* were cocultured with macrophages for 1 h. Phagocytic cells (macrophages containing at least one internalized yeast) were examined under an inverted phase-contrast light microscope (Nikon ECLIPSE Ti-S) and phagocytic activities were calculated from at least 10 high-power fields (HPF) using the following formulas.

Formula 1:

$$\begin{array}{l} \text{Percentage of phagocytic cells}=\\ \;(\text{Number of phagocytic cells in each HPF/Total number of macrophages in each HPF)}\times 100. \end{array}$$

Formula 2:

$$\begin{array}{l} \text{Phagocytic index}=\text{Percentage of phagocytic cells in each HPF}\\ \;\times \text{Average number of internalized yeasts per cell}. \end{array}$$

### Knockdown of Vimentin by Small-Interfering RNA (siRNA)

To further validate functional significance of the COM-treated exosomes in immune response, vimentin whose level was significantly increased in the COM-treated exosomes was selected as the target to be knocked down by siRNA technique. Briefly, macrophages (10 × 10^6^ cells/flask) were transfected with 60 pmol of siRNA targeting on vimentin (si-Vimentin) or the controlled siRNA (si-Control) mixed with transfection reagent in the transfection medium (Santa Cruz Biotechnology) according to the manufacturer’s protocol. After 6-h incubation in a humidified incubator with 5% CO_2_ at 37°C, the transfection medium was removed and replaced with complete RPMI 1640 medium supplemented with 10% (v/v) heat-inactivated FBS and the cells were further incubated for 18 h. At 24-h post-transfection, the si-Control-transfected and si-Vimentin-transfected cells were subjected to COM crystal treatment as described earlier in the non-transfected cells (incubated in serum-free medium with or without 100 µg/ml COM crystals for 16 h). Confirmation of vimentin knockdown in the siRNA-transfected macrophages was performed by immunofluorescence staining as described below, whereas the culture supernatants were collected and subjected to exosome isolation as described above.

### Immunofluorescence Staining

After COM treatment, the si-Control-transfected and si-Vimentin-transfected macrophages were adhered on a coverslip, fixed by 4% (v/v) paraformaldehyde/PBS at 25°C for 15 min, and then permeabilized with 0.2% Triton X-100/PBS at 25°C for 15 min. After washing, the cells were incubated at 4°C overnight with mouse monoclonal anti-vimentin antibody (Santa Cruz Biotechnology) (diluted 1:50 in 1% BSA/PBS). After washing, the cells were incubated with corresponding secondary antibody conjugated with Alexa Fluor 488 (Invitrogen) (diluted 1:2,000 in 1% BSA/PBS) at 25°C for 1 h. Finally, the cells were extensively washed with PBS and mounted onto a glass slide using 50% glycerol in PBS. The cells were imaged by using Nikon Eclipse 80i fluorescence microscope (Nikon). Expression level of vimentin was quantitated by measuring mean fluorescence intensity from at least 50 cells in 10 random HPF of each sample using NIS-Elements D V.4.11 software (Nikon).

### Effects of si-Vimentin vs. si-Control on Activities of the COM-Treated Exosomes on Effector Immune/Inflammatory Cells

After COM treatment, exosomes derived from the si-Control-transfected and si-Vimentin-transfected macrophages were isolated. Thereafter, 30 µg of these intact exosomes were incubated with U937 monocytes, Jurkat T-cells, and macrophages (2 × 10^5^ cells/well) for 24 h and the effector cells were subjected to evaluation of their migratory and phagocytic activities as aforementioned.

### Effects of COM-Treated Exosomes and si-Vimentin on Proinflammatory Cytokine Production in the Effector Immune/Inflammatory Cells

To evaluate effects of COM-treated exosomes and si-Vimentin on proinflammatorty cytokine production in the effector immune/inflammatory cells, U937 monocytes (2 × 10^5^ cells/well) were incubated with 30 µg intact exosomes derived from the non-transfected untreated macrophages (controlled exosomes), si-Control-transfected COM-treated macrophages, and si-Vimentin-transfected COM-treated macrophages for 24 h. The culture supernatant was collected, clarified by centrifugation at 300 *g*, and then subjected to indirect ELISA to measure level of IL-8, one of the proinflammatory cytokines produced by the effector immune/inflammatory cells. The sample preparation and ELISA protocols were similar to those used for ELISA measurement of IL-1β as described above (except for primary antibody that was rabbit polyclonal anti-IL-8 antibody (Santa Cruz Biotechnology) instead).

### Statistical Analysis

Statistical analyses were performed using SPSS software version 13.0 (SPSS; Chicago, IL, USA). Comparisons between two sets of data (e.g., controlled exosome vs. COM-treated exosome) were performed by unpaired Student’s *t*-test, whereas multiple comparisons were performed by one-way ANOVA with Tukey’s *post hoc* test. *P*-values less than 0.05 were considered statistically significant.

## Results

### Morphological and Marker Confirmation

**Figure 1:**
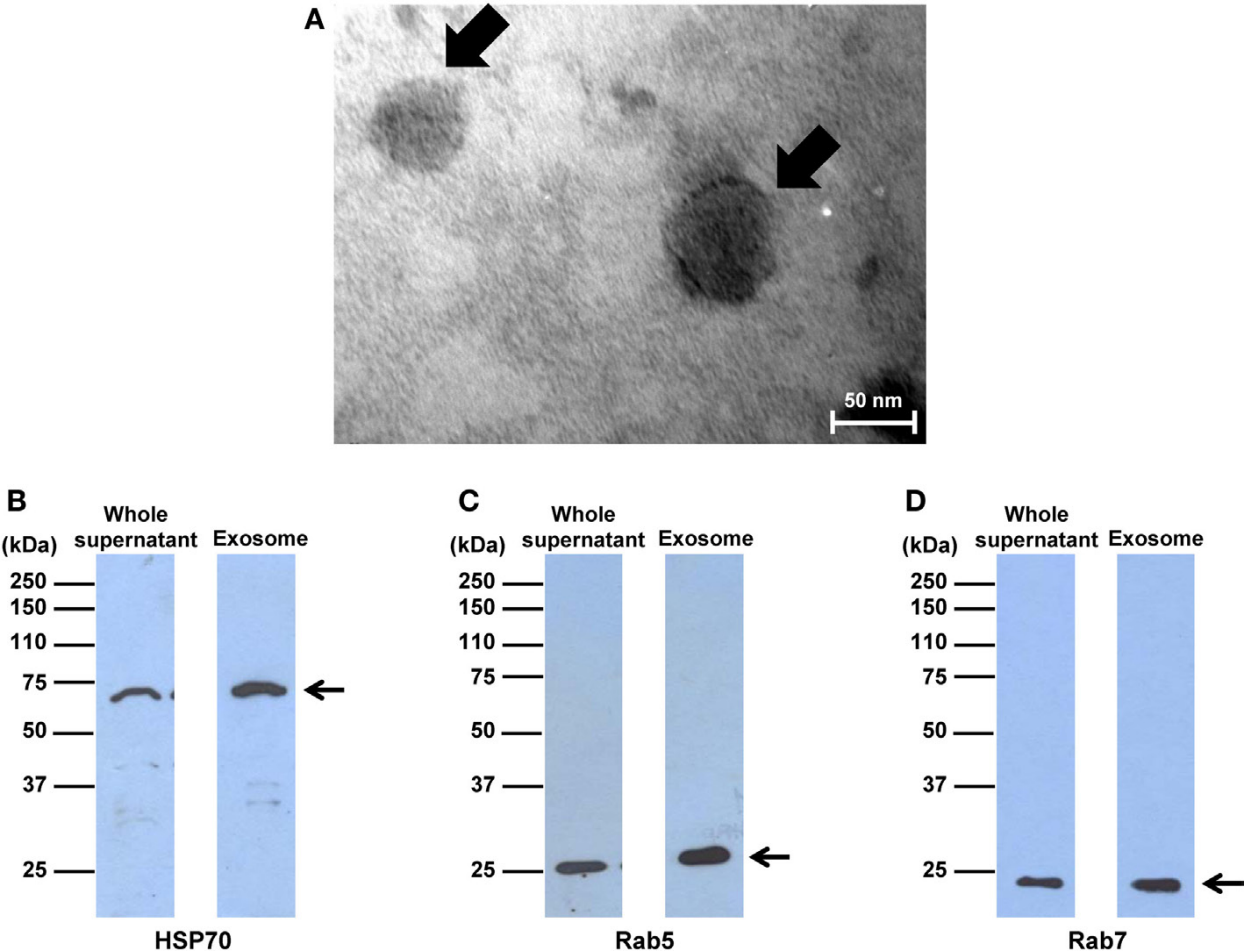
Morphology and markers of exosomes isolated from macrophages. **(A)** Exosomes were isolated from macrophages, negatively stained with uranyl acetate, and imaged by transmission electron microscopy. The arrow indicates typical spherical shape of exosomes at original magnification of 250,000×. **(B–D)** Western blot analyses of exosomal markers, including heat shock protein 70, Rab5, and Rab7, in the whole supernatant compared to the exosome purified fraction (with an equal amount of total protein loaded in each lane). Note that these are the cropped images of the original blots, which are fully shown as Figure S1 in Supplementary Material.

Macrophage exosomes were isolated by microfiltration and differential centrifugation. Their morphology was examined using the negative staining method and visualized by TEM. The results showed membrane-bounded, spherical shape vesicles with a size range of 50–80 nm (Figure 1A), consistent with the typical morphology and size of exosomes reported previously (28). In addition, Western blotting was performed to confirm the expression of exosomal markers. The data showed that levels of HSP70, Rab5, and Rab7, all of which are the typical exosomal markers, were enriched in the exosome purified fraction as compared to the whole supernatant (Figures 1B–D), indicating that isolation of macrophage exosomes was successful.

### Significantly Altered Proteins in Macrophage Exosomes after Exposure to COM Crystals

**Figure 2:**
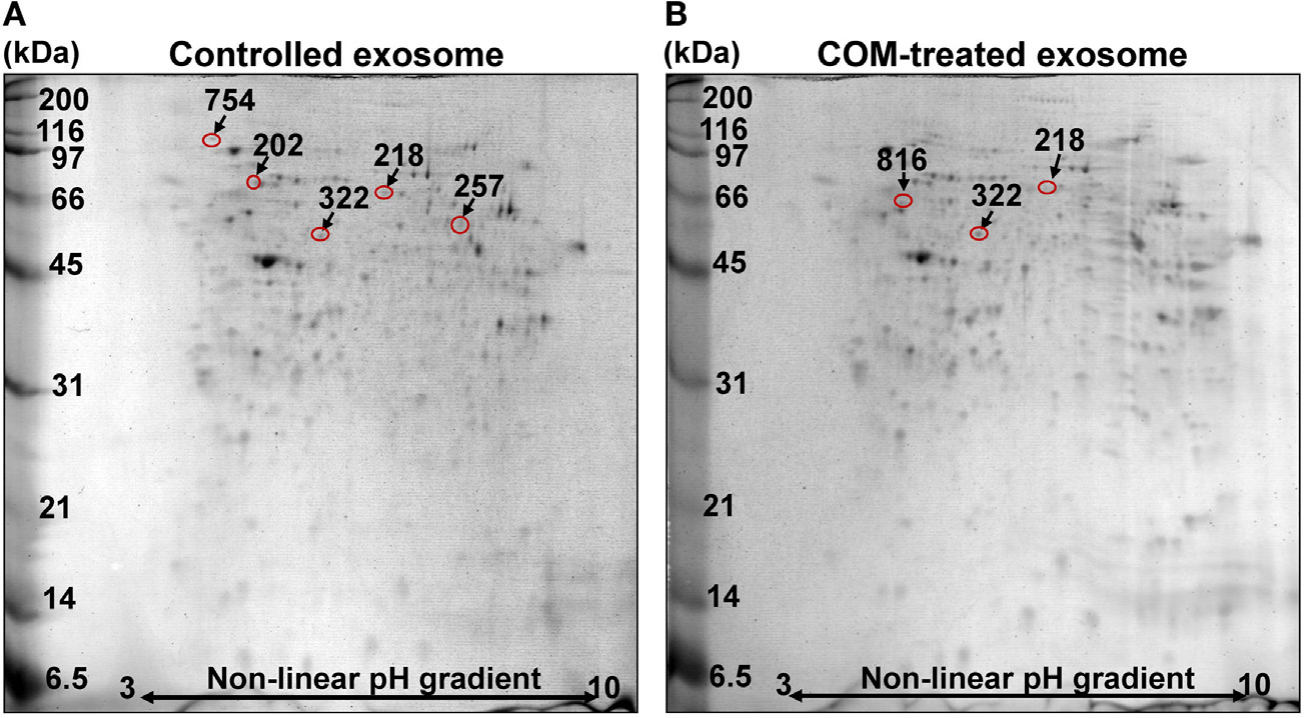
2-D proteome map of differentially expressed proteins. **(A)** Representative 2-D map of the controlled macrophage exosomes. **(B)** Representative 2-D map of exosomes derived from the calcium oxalate monohydrate (COM)-treated macrophages (COM-treated exosomes). Deep Purple fluorescence protein staining, *n* = 5 gels/group, a total of 10 gels were analyzed. The differentially expressed proteins are labeled with numbers that correspond to those indicated in Tables 1 and 2.

Macrophages were treated with or without 100 µg/ml COM crystals for 16 h and their exosomal proteins were then subjected to comparative proteome analysis using 2-DE-based proteomics approach (*n* = 5 gels/group; a total of 10 gels were analyzed). Deep Purple fluorescence protein staining and Image Master 2D Platinum software (GE healthcare) with high stringent criteria for protein spot detection revealed approximately 150–200 protein spots in each 2-D gel (Figure 2). Spot matching, quantitative intensity analysis and statistics revealed six significantly altered protein spots in exosomes derived from COM-treated macrophages (COM-treated exosomes) as compared to the controls (Figure 2). These significantly altered proteins were then successfully identified by nanoLC-ESI-ETD MS/MS analyses (Figure 2), including L-plastin, coronin-like protein, pyruvate kinase, actin-related protein 3 (Arp3), HSP90β, and vimentin (Table 1). All these identified proteins were classified based on their main biological processes and immunological functions using UniProt Knowledgebase (UniProtKB) (http://www.uniprot.org), which is the central hub for collection of functional information of proteins. For biological processes, these included actin filament bundle assembly, actin cytoskeleton organization, ATP biosynthesis processes, actin nucleation, cellular response to unfolded proteins, and intermediate filament organization (Table 2). For immunological functions, most of the identified proteins were involved mainly in immune response, including T-cell activation, T-cell homeostasis, Fc-gamma (Fcγ) receptor pathway mediated phagocytosis, interferon-γ (IFN-γ) regulation, and cell migration and movement (Table 2).

**Table 1:** Summary of significantly altered proteins in macrophage exosomes after exposure to 100 µg/ml COM crystals for 16 h.

Spot no.	Protein name	NCBI ID	MS/MS identification scores	%Cov	No. of distinct/total matched peptides	p*I*	MW (kDa)	Intensity (mean ± SEM)		Ratio (COM-treated/controlled)	*P*-value
								Controlled exosome	COM-treated exosome		
202	L-plastin	gi|62898171	1,215	36	24/37	5.20	70.79	0.1702 ± 0.0724	0.0000 ± 0.0000	0.00	0.047
218	Coronin-like protein	gi|5902134	361	19	10/11	6.25	51.68	0.2537 ± 0.0697	0.0629 ± 0.0562	0.25	0.046
257	Pyruvate kinase isozymes M1/M2 isoform 2	gi|488544468	462	17	11/11	7.96	58.43	0.1466 ± 0.0634	0.0000 ± 0.0000	0.00	0.046
322	Actin-related protein 3 isoform 1	gi|5031573	512	22	10/12	5.61	47.80	0.3521 ± 0.0297	0.5521 ± 0.0452	1.57	0.006
754	HSP90-beta	gi|306891	87	11	9/9	4.97	85.58	0.2632 ± 0.0743	0.0000 ± 0.0000	0.00	0.008
816	Vimentin	gi|340219	730	39	20/22	5.03	53.74	0.0000 ± 0.0000	1.1192 ± 0.3448	#DIV/0	0.043

*%Cov = %Sequence coverage [(number of the matched residues/total number of residues in the entire sequence) × 100%]*.

*#DIV/0 = Divided by zero*.

*NCBI, National Center for Biotechnology Information*.

**Table 2:** Biological process and immunological function of the significantly altered proteins in COM-treated exosomes.

Spot no.	Protein name	Biological process	Immunological function	Alteration in COM-treated exosomes
202	L-plastin	Actin filament bundle assembly	T-cell activation involved in immune response	Decreased
218	Coronin-like protein	Actin cytoskeleton organization	T-cell homeostasis	Decreased
257	Pyruvate kinase isozymes M1/M2 isoform 2	ATP biosynthesis process	–	Decreased
322	Actin-related protein 3 isoform 1	Actin nucleation	Fcγ receptor pathway mediated phagocytosis	Increased
754	HSP90-beta	Cellular response to unfold proteins	IFN-γ regulation	Decreased
816	Vimentin	Intermediate filament organization	Cell migration and movement	Increased

### Validation of the Proteome Data by Western Blotting

**Figure 3:**
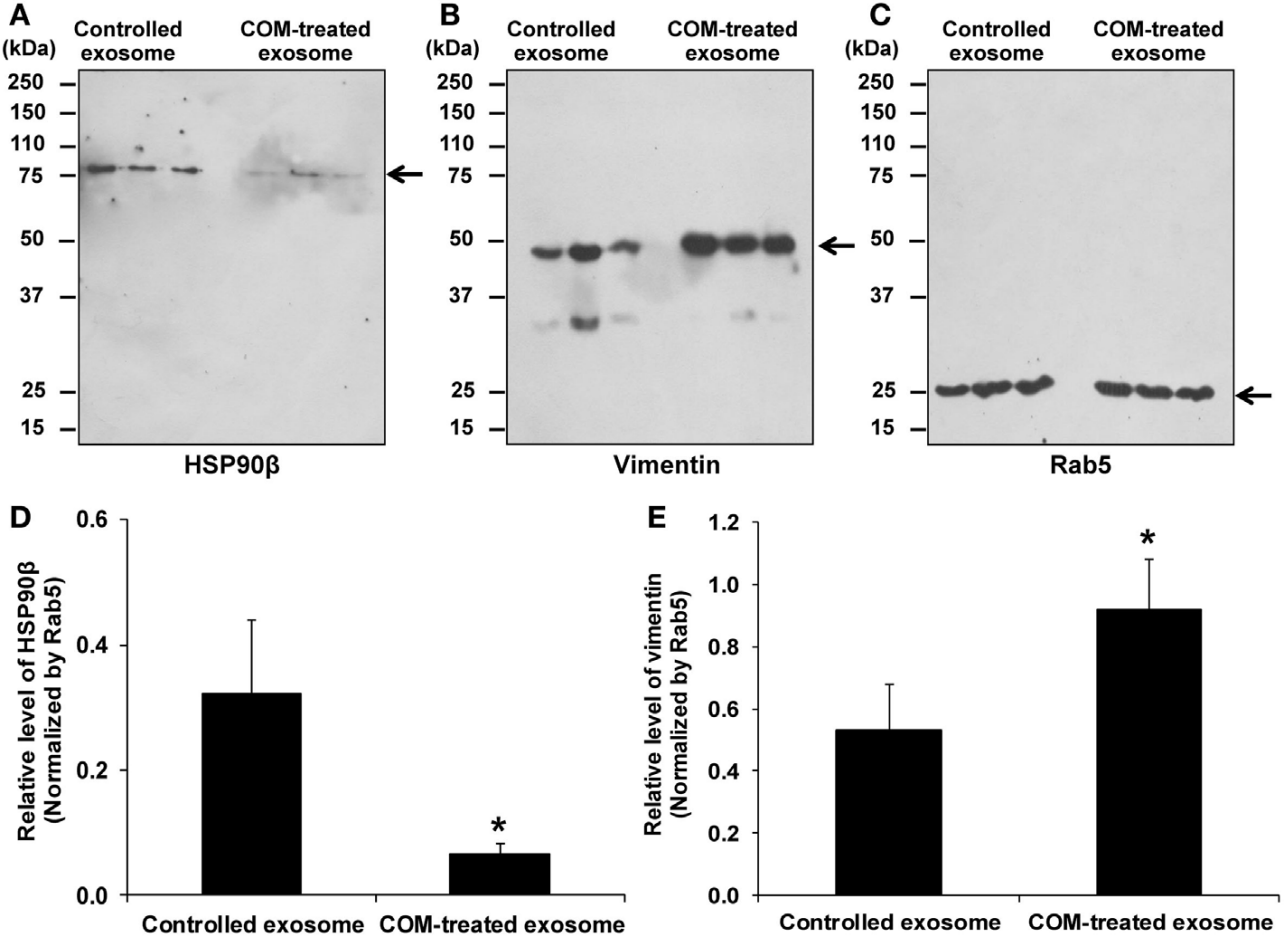
Validation of the proteome data by Western blot analysis. **(A)** The decreased level of HSP90β in exosomes derived from the COM-treated macrophages (COM-treated exosomes). **(B)** The increased level of vimentin in the COM-treated exosomes. **(C)** Rab5 served as the loading control. **(D)** Normalized level of HSP90β. **(E)** Normalized level of vimentin. Each bar represents mean ± SD of the data obtained from three independent biological replicates. *P < 0.05 vs. controlled exosomes.

Western blot analysis was performed to validate the proteome data of two selected representative proteins with decreased and increased levels, respectively. The data demonstrated that the decreased level of HSP90β and increased level of vimentin in COM-treated exosomes as determined by 2-DE-based proteome analysis could be confirmed by Western blot analysis using Rab5, an exosomal marker, as the loading control to normalize (Figure 3).

### Effects of COM Crystals on Inflammasome Activation

**Figure 4:**
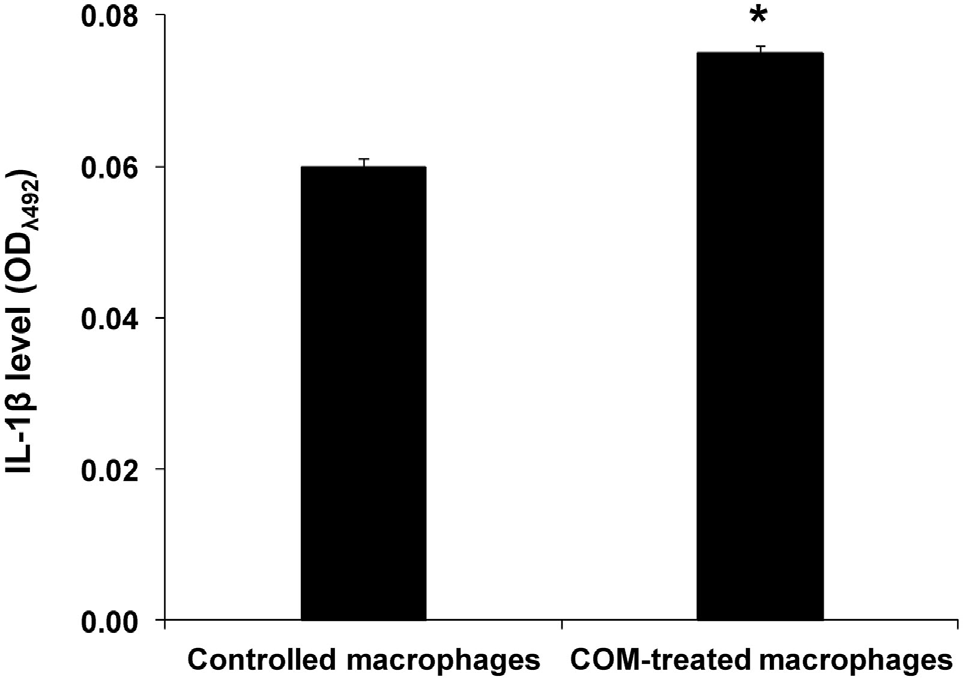
Effects of calcium oxalate monohydrate (COM) crystals on inflammasome activation. Interelukin-1β level was measured by ELISA in culture supernatants of macrophages with or without COM crystal treatment. Each bar represents mean ± SD of the data obtained from three independent biological replicates. *P < 0.05 vs. control.

To evaluate the inflammatory response induced by COM crystal treatment, activation of inflammasome was evaluated by measuring level of IL-1β, one of the markers of inflammasome activation, in culture supernatants of the COM-treated vs. controlled macrophages. ELISA revealed significant increase in IL-1β production in the COM-treated macrophages, suggesting that inflammasome was activated by COM crystal treatment (Figure 4).

### Effects of COM-Treated vs. Controlled Exosomes on Monocyte and T-Cell Migration

**Figure 5:**
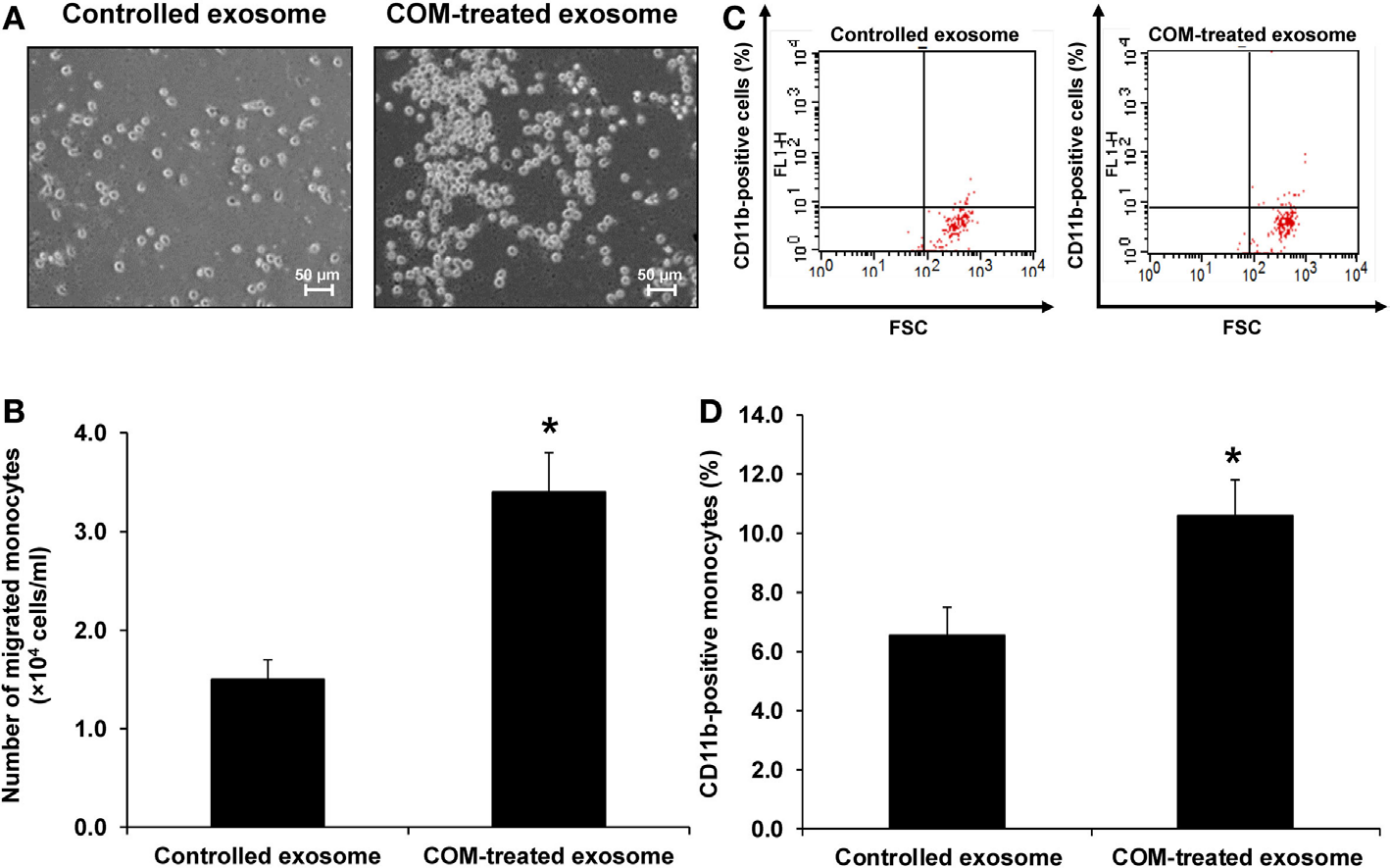
Effects of calcium oxalate monohydrate (COM)-treated vs. controlled exosomes on monocyte migration and activation. **(A)** The migrated monocytes at the lower chamber of transwell were examined and imaged using an inverted phase-contrast light microscope with an original magnification of 200×. **(B)** The migrated monocytes were counted from at least 10 low-power fields (LPF). **(C)** The representative dot plot of flow cytometric data in each group to quantitate the CD11b-positive migrated monocytes (FSC, forward scatter, indicating cell size). **(D)** The percentage the CD11b-positive cells. Each bar represents mean ± SD of the data obtained from three independent biological replicates. *P < 0.05 vs. controlled exosomes.

**Figure 6:**
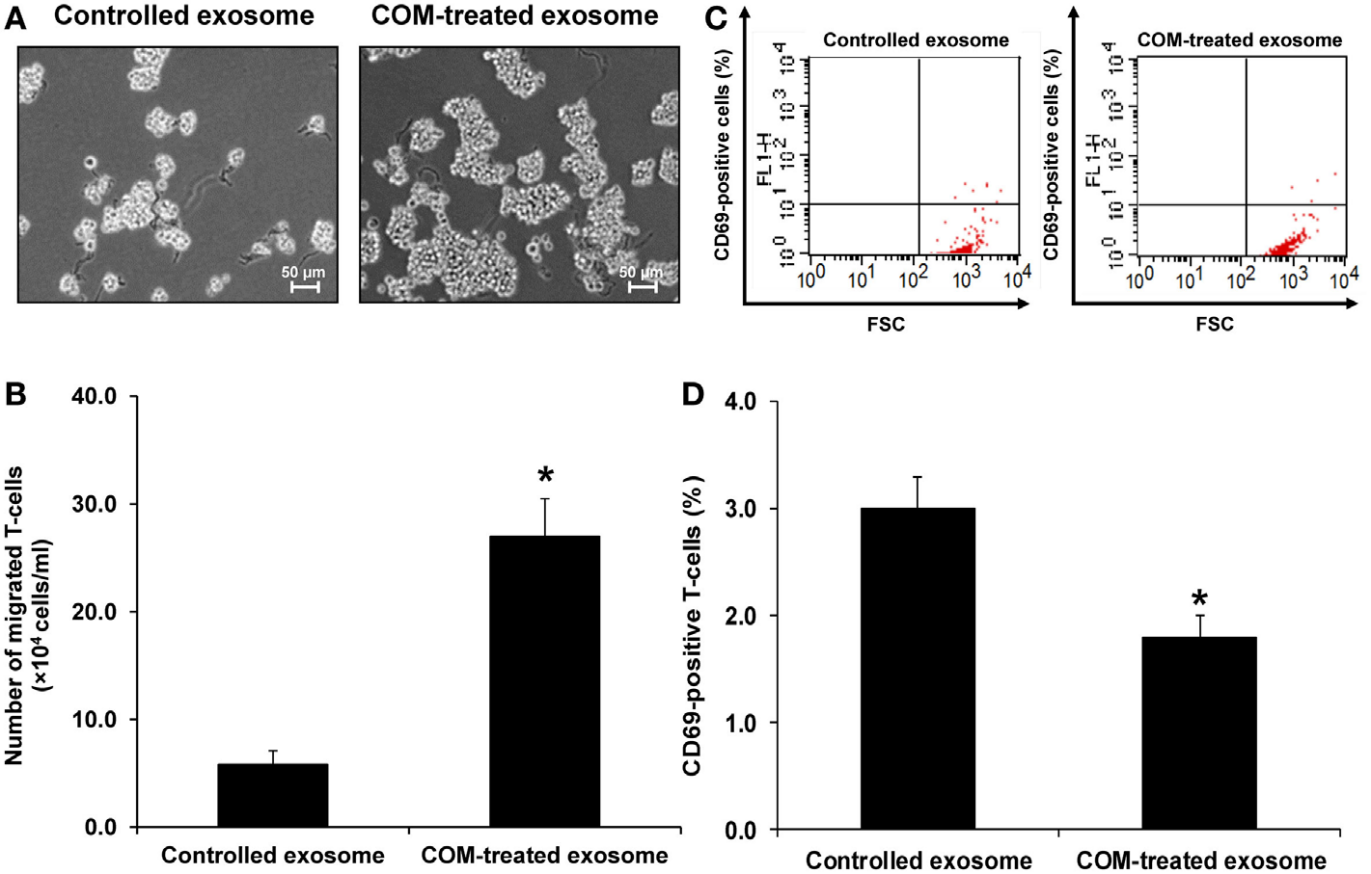
Effects of calcium oxalate monohydrate (COM)-treated vs. controlled exosomes on T-cell migration and activation. **(A)** The migrated T-cells at the lower chamber of transwell were examined and imaged using an inverted phase-contrast light microscope with an original magnification of 200×. **(B)** The migrated T-cells were counted from at least 10 low-power fields (LPF). **(C)** The representative dot plot of flow cytometric data in each group to quantitate the CD69-positive migrated T-cells (FSC, forward scatter, indicating cell size). **(D)** The percentage the CD69-positive cells. Each bar represents mean ± SD of the data obtained from three independent biological replicates. *P < 0.05 vs. controlled exosomes.

Because most of the altered proteins were involved in immune response and vimentin (one of the proteins involving in cell migration/movement) was markedly increased in the COM-treated exosomes, we thus speculated that the COM-treated exosomes might affect migration of monocytes and T-cells. Our hypothesis was addressed by evaluation of the effects of COM-treated vs. controlled exosomes on monocyte and T-cell migration. The migration assay was performed using transwell and numbers of the migrated monocytes and T-cells were counted after incubation with these differential exosomes for 24 h. The results showed marked increases in numbers of migrating monocytes (Figures 5A,B) and T-cells (Figures 6A,B) after induction with COM-treated exosomes as compared to the controlled exosomes.

### Effects of COM-Treated vs. Controlled Exosomes on Monocyte and T-Cell Activation

Activation of monocytes and T-cells was evaluated by measuring CD11b-positive monocytes and CD69-positive T-cells, respectively. Flow cytometric analysis revealed that number of the CD11b-positive monocytes was significantly increased (Figures 5C,D), whereas that of the CD69-positive cells was significantly decreased (Figures 6C,D) by the COM-treated exosomes.

### Effects of COM-Treated vs. Controlled Exosomes on Macrophage Phagocytic Activity

**Figure 7:**
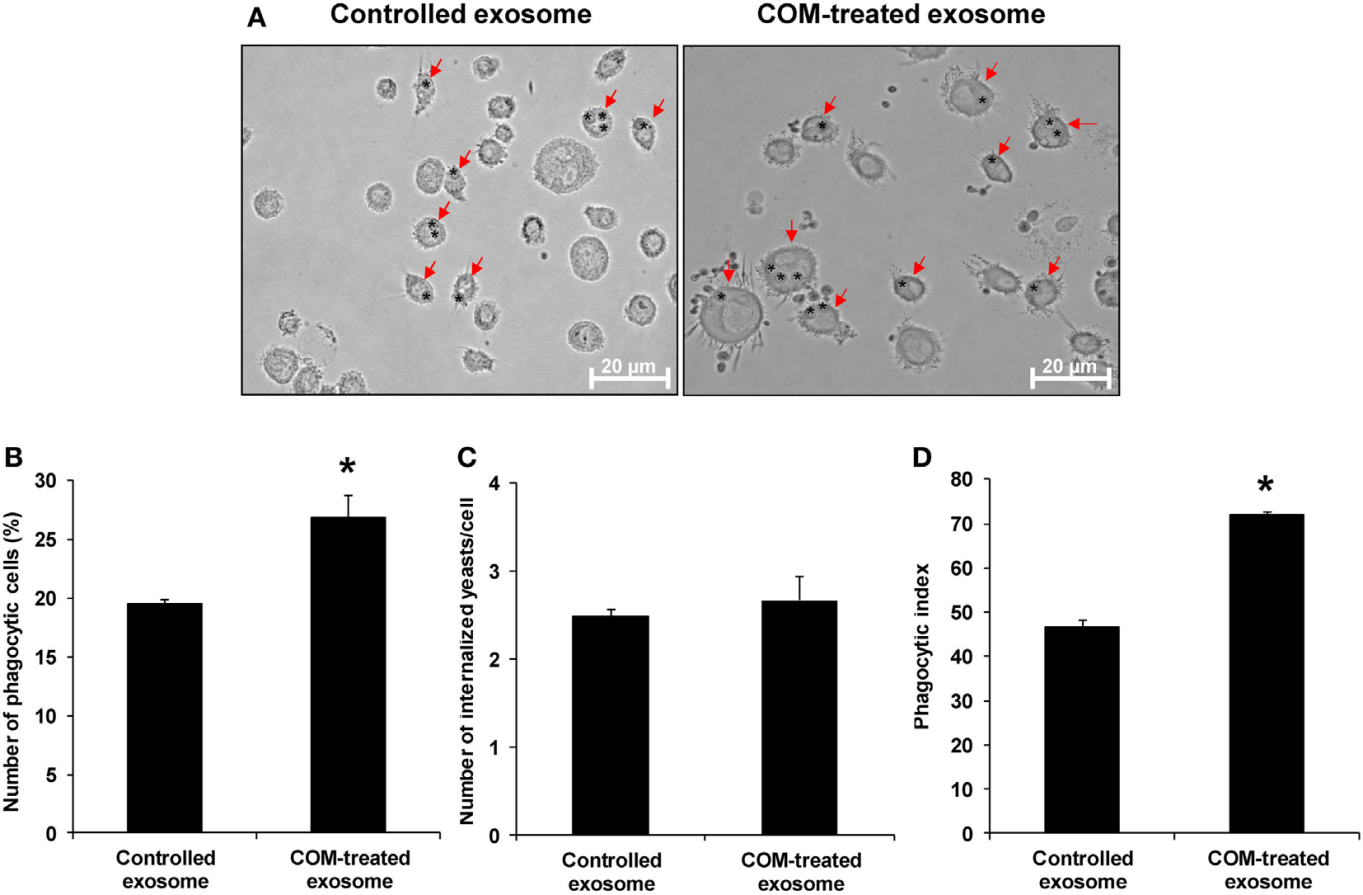
Effects of calcium oxalate monohydrate (COM)-treated vs. controlled exosomes on macrophage phagocytic activity. Macrophages were incubated with intact controlled exosomes or COM-treated exosomes for 24 h followed by incubation with *Saccharomyces cerevisiae* for 1 h. **(A)** The phagocytic cells (indicated with arrows), representing macrophages with at least one internalized yeast (indicated with asterisks), were examined under an inverted phase-contrast light microscope with the original magnification of 400×. Phagocytic activities were calculated from at least 10 high-power fields to determine percentage of the phagocytic cells **(B)**, number of the internalize yeasts per cell **(C)**, and phagocytic index **(D)** (see formulas in Section “Materials and Methods”). Each bar represents mean ± SD of the data obtained from three independent biological replicates. *P < 0.05 vs. controlled exosomes.

Because Arp3, a protein involving in Fcγ receptor pathway mediated phagocytosis, was significantly increased in COM-treated exosomes, we thus hypothesized that the COM-treated exosomes might affect macrophage phagocytic activity. Our hypothesis was addressed by evaluation of the effects of COM-treated vs. controlled exosomes on macrophage phagocytic activity. The results showed that number of the phagocytic cells and phagocytic index of macrophages were significantly increased after the cells were exposed to the COM-treated exosomes as compared to the controlled exosomes (Figure 7).

### Effects of si-Vimentin vs. si-Control on Activities of the COM-Treated Exosomes on Effector Immune/Inflammatory Cells

**Figure 8:**
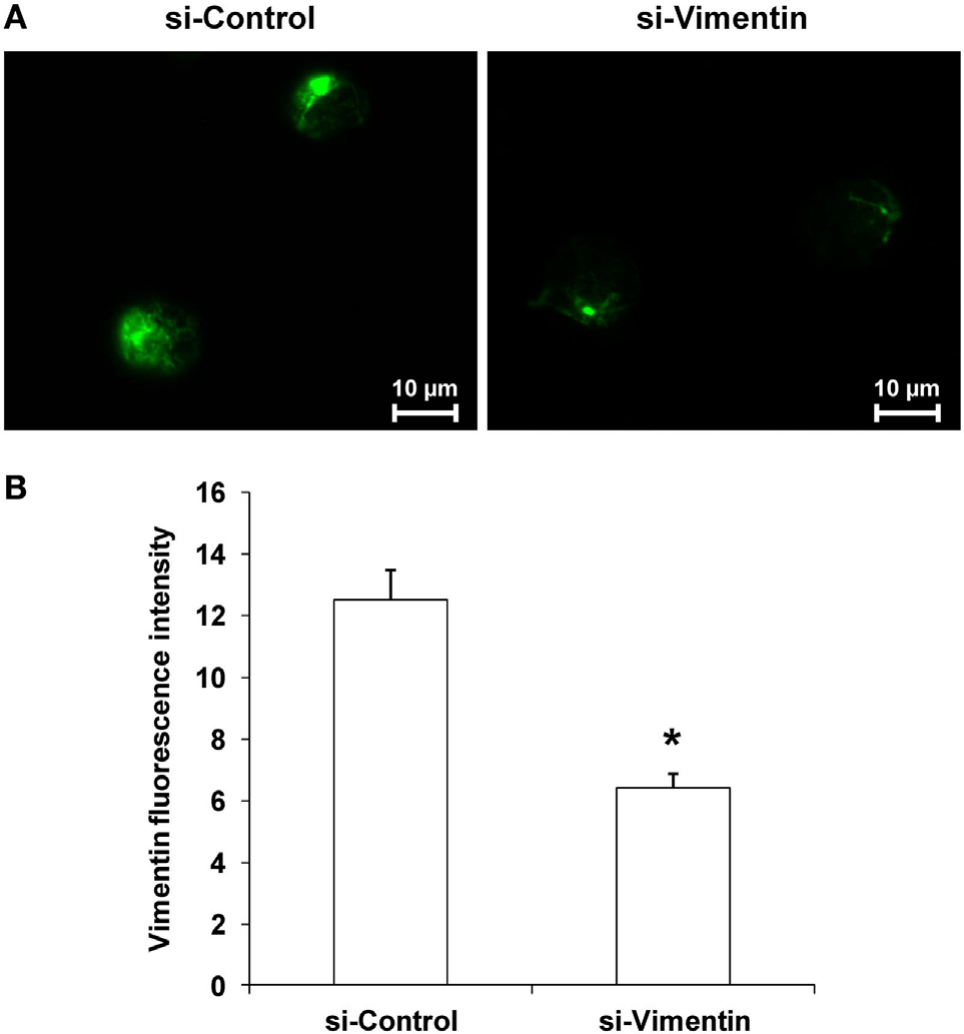
Efficacy of knockdown of vimentin by small-interfering RNA (siRNA). **(A)** Immunofluorescence staining of vimentin in the siRNA targeting on vimentin-transfected vs. si-Control-transfected macrophages after calcium oxalate monohydrate treatment. **(B)** Mean fluorescence intensity of vimentin was measured from at least 10 high-power fields. Each bar represents mean ± SD of the data obtained from three independent biological replicates. *P < 0.05 vs. si-Control.

To validate functional relevance of the COM-treated exosomes in immune response, vimentin whose level was significantly increased in the COM-treated exosomes was knocked down by siRNA technique. The efficacy of siRNA targeting on vimentin (si-Vimentin) was confirmed by immunofluorescence staining, which showed markedly decreased level of vimentin in the si-Vimentin-transfected COM-treated macrophages as compared to the si-Control-transfected COM-treated macrophages (Figure 8).

**Figure 9:**
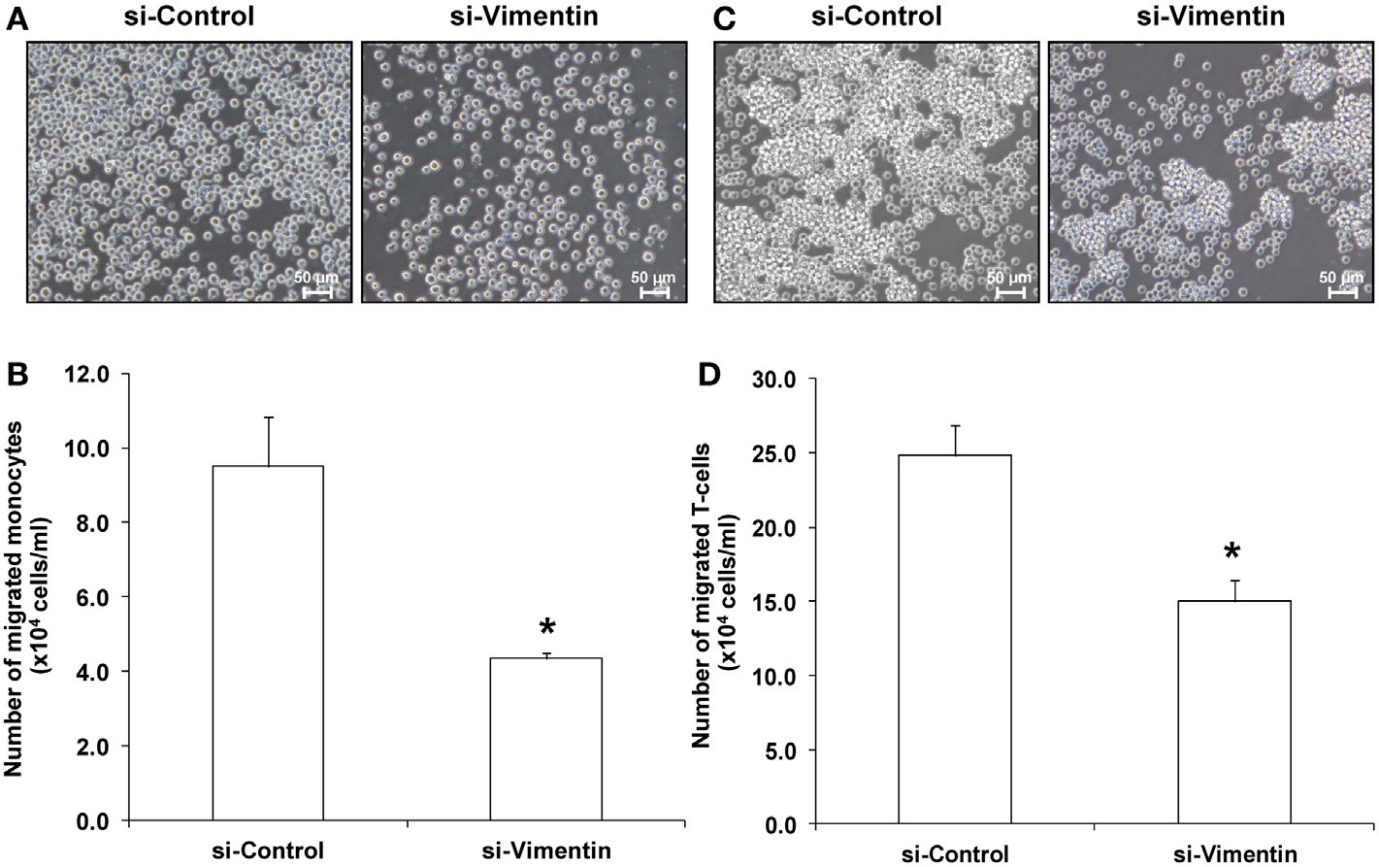
Effects of small-interfering RNA targeting on vimentin vs. si-Control on activities of the calcium oxalate monohydrate-treated exosomes on monocyte and T-cell migration. This experiment was performed using transwell similar to that of the non-transfected cells. The migrated monocytes **(A)** and T-cells **(C)** at the lower chamber of transwell were examined and imaged using an inverted phase-contrast light microscope with an original magnification of 200×. The migrated monocytes **(B)** and T-cells **(D)** were counted from at least 10 low-power fields. Each bar represents mean ± SD of the data obtained from three independent biological replicates. *P < 0.05 vs. si-Control.

**Figure 10:**
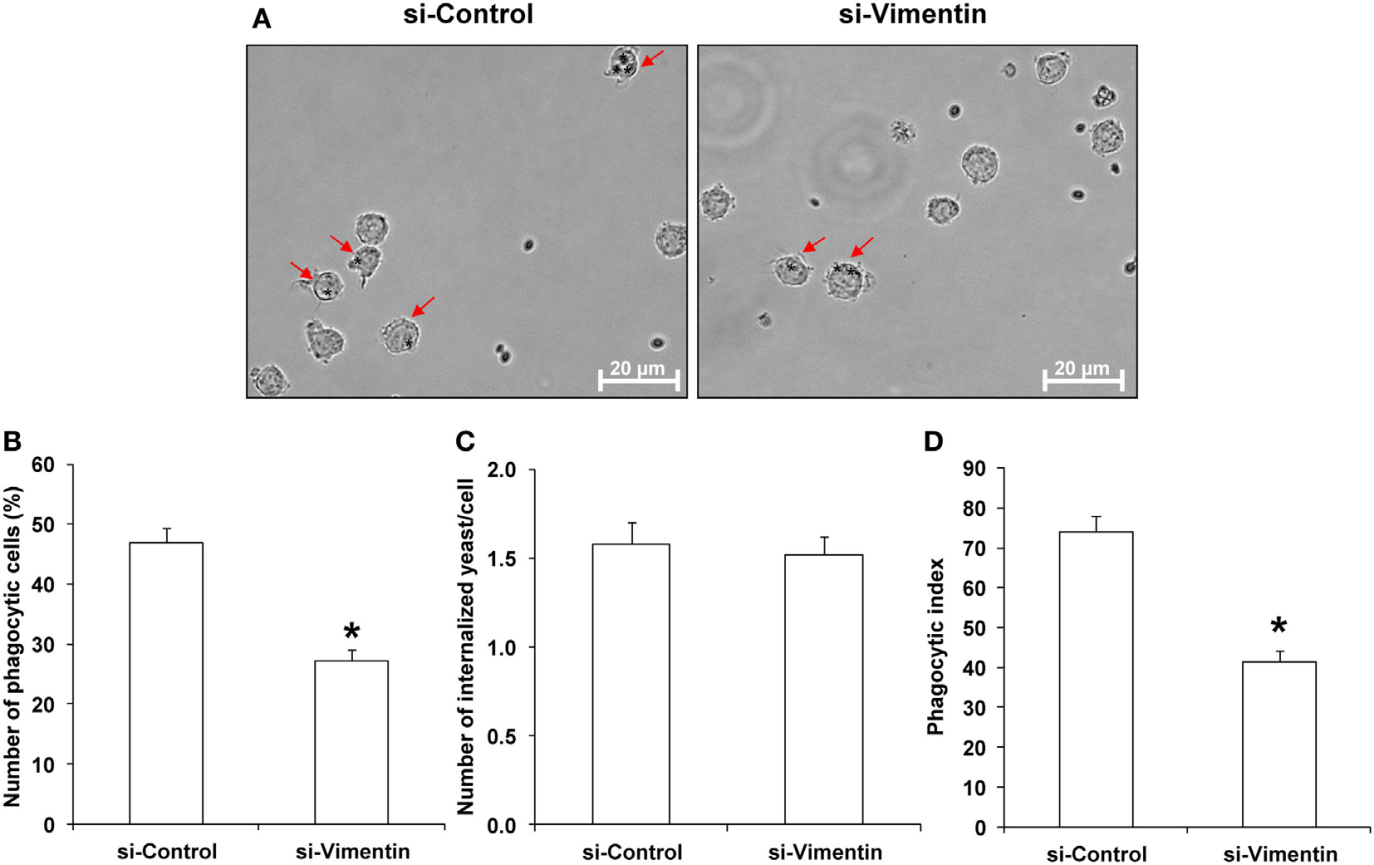
Effects of small-interfering RNA targeting on vimentin (si-Vimentin) vs. si-Control on activities of the calcium oxalate monohydrate (COM)-treated exosomes on macrophage phagocytic activity. Macrophages were incubated with intact exosomes derived from si-Control-transfected COM-treated or si-Vimentin-transfected COM-treated macrophages for 24 h followed by incubation with *Saccharomyces cerevisiae* for 1 h. **(A)** The phagocytic cells (indicated with arrows), representing macrophages with at least one internalized yeast (indicated with asterisks), were examined under an inverted phase-contrast light microscope with the original magnification of 400×. Phagocytic activities were calculated from at least 10 high-power fields to determine percentage of the phagocytic cells **(B)**, number of the internalize yeasts per cell **(C)**, and phagocytic index **(D)** (see formulas in Section “Materials and Methods”). Each bar represents mean ± SD of the data obtained from three independent biological replicates. *P < 0.05 vs. si-Control.

Exosomes derived from these si-Vimentin-transfected COM-treated and si-Control-transfected COM-treated macrophages were then isolated and subjected to functional assays to evaluate migratory and phagocytic activities of the effector immune/inflammatory cells. The data showed that the COM-treated exosomes derived from the si-Vimentin-transfected macrophages caused significant decreases in migratory activities of both monocytes and T-cells as compared to the COM-treated exosomes derived from the si-Control-transfected macrophages (Figure 9). In addition, macrophages incubated with the COM-treated exosomes derived from the si-Vimentin-transfected cells had significantly fewer numbers of phagocytic cells and less phagocytic index than when they were incubated with the COM-treated exosomes derived from the si-Control-transfected cells (Figure 10).

### Effects of COM-Treated Exosomes and si-Vimentin on Proinflammatory Cytokine Production in the Effector Immune/Inflammatory Cells

**Figure 11:**
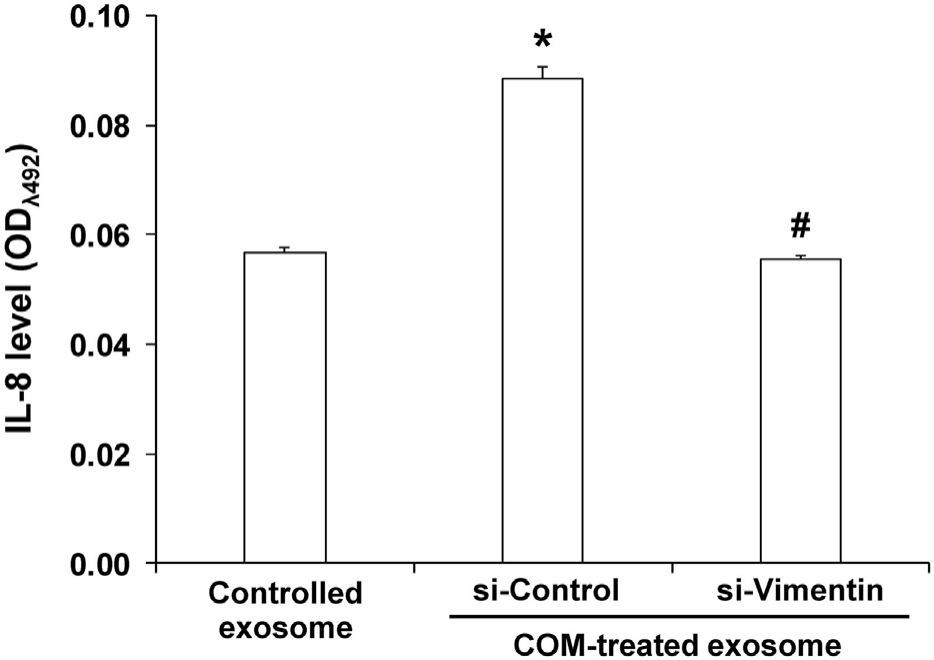
Effects of calcium oxalate monohydrate (COM)-treated exosomes and small-interfering RNA targeting on vimentin (si-Vimentin) on proinflammatory cytokine production in the effector immune/inflammatory cells. U937 monocytes were incubated with intact exosomes derived from the non-transfected untreated (controlled exosome), si-Control-transfected COM-treated, or si-Vimentin-transfected COM-treated macrophages for 24 h. The culture supernatants were collected and subjected to indirect ELISA to measure level of interleukin-8, one of the proinflammatory cytokines produced by the effector immune/inflammatory cells. Each bar represents mean ± SD of the data obtained from three independent biological replicates. *P < 0.05 vs. controlled exosome; ^#^*P* < 0.05 vs. si-Control-transfected COM-treated exosome.

To evaluate effects of COM-treated exosomes and si-Vimentin on proinflammatory cytokine production in the effector immune/inflammatory cells, ELISA was performed to measure level of IL-8, one of the proinflammatory cytokines, produced from U937 monocytes incubated with exosomes derived from the non-transfected untreated macrophages (controlled exosomes), si-Control-transfected COM-treated macrophages, and si-Vimentin-transfected COM-treated macrophages. The data revealed significantly increased level of IL-8 produced by the cells incubated with the COM-treated exosomes derived from the si-Control-transfected macrophages, whereas si-Vimentin successfully restored IL-8 to the basal level (comparable to the cells incubated with the controlled exosomes) (Figure 11).

## Discussion

Exosomes are originated from the internalized vesicles *via* endocytosis that subsequently form multivesicular bodies (MVB) (29). They are secreted to extracellular milieu *via* exocytosis pathway by fusing MVB with plasma membranes (30). Exosomes contain several types of biomolecules, including mRNAs, microRNAs, proteins and lipids, which reflect their diverse biological functions (31). From their origination, exosomes are commonly enriched with proteins associated with MVB biogenesis, transport and fusion (30). In addition, they are also enriched with intregrins (CD81 and CD82), tetraspanins (CD9 and CD63), chaperones (HSP70 and HSP90), and major histocompatibility complex class I and class II (32). For intercellular communications, exosomes shuttle their biomolecules to the target cells by three major mechanisms, including receptor–ligand interaction, direct fusion with plasma membranes, and endocytosis (30).

Macrophage exosomes have been demonstrated to possess immune functions in several diseases, including host–pathogen interactions and cancers (30). For host–pathogen interactions, *in vitro* studies have shown that macrophages with intracellular pathogens secrete greater amounts of exosomes as compared to the uninfected cells. Exosomes derived from these infected macrophages activate naive macrophages through tumor necrosis factor-α and IL-12, and subsequently recruit monocytes and neutrophils into the inflammatory sites (33, 34). In cancers, macrophage-derived exosomes can promote cancer cell invasion and metastasis. The proteome data has demonstrated that these exosomes have increased levels of matrix metalloproteinase (MMP) and cathepsins, which can cleave extracellular matrix facilitating tumor dissemination (35).

In kidney stone disease, infiltration of macrophages in the renal interstitium can promote chronic inflammation, leading to chronic kidney disease (1–3). Macrophages secrete several types of biomolecules in response to CaOx crystals deposited in renal interstitium, including ROS, chemokines, proinflammatory cytokines, and fibrogenic factors that subsequently stimulate the inflammatory processes and provoke tubulointerstitial damage (10–12). These secretory products may also play important autocrine and/or paracrine roles in the renal interstitial milieu. In addition, interstitial CaOx crystal deposition can then activate mononuclear phagocytes (i.e., dendritic cells and macrophages) to secret IL-1β through NLRP3/ASC/caspase-1-dependent pathway, causing renal inflammation in kidney stone disease (15). These findings indicate that CaOx crystals are also involved in activation of inflammasome, the multiprotein complex that plays crucial role in innate immunity (15). Likewise, infection and cellular stress can enhance inflammasome activation in the activated macrophages as indicated by redistribution and spatial organization of ASC (apoptotic speck-like protein containing a CARD) to the cytoplasm, followed by assembly of inflammasome components, including Nod-like receptors (NLR) and caspase-1 in the perinuclear space, which is necessary for inflammasome function such as maturation of IL-1β and IL-18 for further inflammatory signaling. In contrast, primary localization of ASC and caspase-1 in the nucleus is commonly observed in the resting monocytes/macrophages (36). Consistent with the previous reports, we demonstrated herein that COM crystals could induce inflammasome activation in macrophages, leading to the increased level of IL-1β, one of the markers for inflammasome activation, in culture supernatant (Figure 4). In addition, we have demonstrated for the first time that COM crystals could induce changes in proteins expressed in exosomes isolated from macrophages and these altered exosomal proteins were involved in several immune functions (Figure 2; Tables 1 and 2).

Vimentin is the most abundant intermediate filament that stabilizes cellular architecture (37). In immune cells, vimentin can be secreted from the activated monocytes, macrophages, and neutrophils and is responsible for activating cell migration, proinflammatory signaling, and oxidative burst (38). In cancers, exosomes derived from macrophages can induce cytoskeletal rearrangement by transferring vimentin-containing exosomes, which further stimulate metastasis of the cancer cells through Wnt signaling pathway (39, 40). Herein, our proteome data showed that vimentin was markedly increased in the COM-treated exosomes that could be confirmed by Western blot analysis. Note that vimentin band at low molecular mass (approximately 30 kDa) was observed in some samples, especially in the controlled exosomes (Figure 3B). In monocyte-derived macrophages, it was possible that vimentin could be degraded during sample processing or cleaved by proteases and then secreted during differentiation process (41). Alternatively, it could be the protein kinase C-dependent phosphorylated form of vimentin that was secreted by activated macrophages (38).

From the proteome data, we hypothesized that the COM-treated exosomes might promote migratory activity of other immune cells in the renal interstitium. Accordingly, migration assay was performed to evaluate migratory activities of monocytes and T-cells exposed to COM-treated vs. controlled exosomes. The functional data confirmed that the COM-treated exosomes dramatically enhanced monocyte and T-cell migration (Figures 5A,B and 6A,B). Monocytes are important responder cells in the renal interstitium to develop chronic inflammation in kidney stone disease (42, 43). Under inflammatory response, monocytes are stimulated to enhance their immune functions. Our functional data clearly demonstrated the activation of monocytes by COM-treated exosomes as evidenced by an increase of CD11b, which is a marker for monocyte activation (44), on their surfaces (Figures 5C,D). In contrast, we found the decreases of L-plastin and coronin-like protein in the COM-treated exosomes. L-plastin is the actin-bundling protein that is exclusively found in leukocytes, i.e., macrophages, lymphocytes, neutrophils, and other granulocytes (40, 45). This protein consists of two tandem repeated actin-binding domains, which are responsible for F-actin bundling and rearrangement (46). Interestingly, L-plastin has been reported to specifically induce T-lymphocyte activation (47). Likewise, coronin-like protein is an actin and microtubule binding protein that plays pivotal role in T-cell activation (48). Their decreases implicated a reduction of T-cell activation. Our functional data clearly showed the significant decrease of CD69, a marker for T-cell activation (49), on surfaces of T-cells exposed to the COM-treated exosomes confirming that T-cell activation was reduced by the COM-treated exosomes (Figures 6C,D).

Furthermore, we also observed significant increase in level of Arp3 isoform1 in the COM-treated exosomes. Arp3 is a member of Arp2/3 complex, which is localized on cell surfaces and is essential for filopodia and lamellipodia structure (50). Arp2/3 complex is important for cell motility and phagosome formation that are the critical steps for phagocytic activity of phagocytes (51). Our functional data showed clear evidence that the COM-treated exosomes had an autocrine function by activation of phagocytic activity of macrophages (Figure 7). Therefore, the increased level of Arp 3 in the COM-treated exosomes might be responsible for such autocrine effect.

Finally, to further confirm the functional relevance of our proteome findings in immune response, vimentin was knocked down by siRNA technique. The data have confirmed that macrophage exosomal vimentin played significant roles in immune response to COM crystals and were involved in proinflammatory cytokine production, monocyte and T-cell migration, and phagocytic activity of macrophages (Figures 9–11). Regarding its role in immune response, a recent study has revealed that vimentin can be secreted by activated macrophages in response to either pro- or anti-inflammatory cytokines. In response to pathogens, secreted vimentin has been implicated in producing oxidative metabolites that are essential for effective bacterial killing by the activated macrophages (38). In addition, vimentin has been reported as a chemoattractant for monocyte migration, consistent to our findings. Interestingly, truncated vimentin generated by leukolysin (also known as MMP25) can enhance phagocytic activity of macrophages (52). These findings support the role of the exosomal vimentin in recruitment of immune cells and enhancement of phagocytic activity of macrophages observed in our study. Nevertheless, the knowledge on biological roles of extracellular vimentin in immune function is currently limited. Therefore, molecular mechanisms of exosomal vimentin in immunology deserve further investigations.

Although this study was quite convincing to clarify significant immune functions of the COM-treated exosomes related to inflammatory response in kidney stone disease, some technical limitations should be mentioned. First, using the 2-DE-based proteomics approach, a relatively small number of altered proteins were identified. Additionally, some of the identified altered proteins were detectable only in one group, but were under the detectability limit in the other group by using this approach (Table 1). Actually, they were not really absent in such group as in cases of HSP90β and vimentin, which could not be detected by 2-DE in the COM-treated and controlled exosomes, respectively, whereas they were detectable in both groups by Western blotting but with significant changes in their levels (Figure 3). Moreover, 2-DE-based approach has another limitation in resolving membrane or highly hydrophobic proteins, which are the major constituents on exosomal surfaces. Therefore, using gel-free and other more sensitive proteomics approaches would overcome such limitations and yield a wider image of significant impact of macrophage exosomes in pathogenic mechanisms of kidney stone disease.

Second, there was a difference in number of migrated monocytes induced by exosomes derived from the COM-treated macrophages in the former experiment (Figure 5B) as compared to that induced by exosomes derived from the si-Control-transfected COM-treated macrophages in the latter to validate the functional relevance of macrophage exosomal vimentin in the immune response to COM crystals (Figure 9B). This difference was most likely due to inter-assay variations (particularly from different batches of U937 cell aliquots used and different lots of PMA employed for macrophage derivatization). Nevertheless, each of these experiments had its own corresponding control. Therefore, the functional relevance of macrophage exosomes should not be hampered by these common variations. Similar phenomenon was observed for the percentage of phagocytic cells induced by exosomes derived from the COM-treated macrophages in the former experiment (Figure 7B) as compared to that induced by exosomes derived from the si-Control-transfected COM-treated macrophages in the latter (Figure 10B). However, the consistency in phagocytic index in both sets of experiments on different occasions (Figures 7D and 10D, respectively) might be able to strengthen our claim.

Finally, it should be noted that only vimentin was selected for functional validation of the immunological roles of macrophage exosomes in response to COM crystals by si-RNA technique. Other altered proteins reported in Tables 1 and 2 might also play significant roles in such immune response as well. Manipulation of their expression (by knockdown and/or overexpression methods) would provide more lines of evidence to convince the crucial roles of macrophage exosomes in progressive interstitial inflammation in kidney stone pathogenic mechanisms.

In summary, we have reported herein changes in macrophage exosomal proteins after exposure to COM crystals. These altered exosomal proteins were involved mainly in immune response with possible autocrine and/or paracrine effects. Specifically, the COM-treated exosomes induced proinflammatory cytokine production, increased monocyte and T-cell migration, and promoted monocyte activation while reduced T-cell activation. In addition, the COM-treated exosomes enhanced phagocytic activity of macrophages. Moreover, our present study has demonstrated for the first time that the macrophage exosomal vimentin played significant roles in the immune response to COM crystals, although the significant roles of other exosomal proteins could not be entirely excluded. Taken together, these findings provided some implications to the immune response during kidney stone pathogenesis *via* exosomal pathway of macrophages after exposure to COM crystals.

## Author Contributions

NS, RK, AN, and VT designed research. NS, RK, and AN performed experiments. NS, RK, AN, and VT analyzed data. All authors wrote, reviewed, and approved the manuscript.

## Conflict of Interest Statement

The authors declare that the research was conducted in the absence of any commercial or financial relationships that could be construed as a potential conflict of interest.
