# Supplementary material for: Roles of Macrophage Exosomes in Immune Response to Calcium Oxalate Monohydrate Crystals
Source: Front Immunol. 2018 Feb 27;9:316. doi: 10.3389/fimmu.2018.00316 (PMC5835051; doi:10.3389/fimmu.2018.00316)
Supplement: Supplementary file 1 [file Image_1.PDF]

SUPPORTING INFORMATION

Roles of macrophage exosomes in immune response to calcium oxalate monohydrate crystals

*Nilubon Singhto, Rattiyaporn Kanlaya, Angkhana Nilnumkhum, and Visith Thongboonkerd**
*(*Correspondence to: thongboonkerd@dr.com (or) vthongbo@yahoo.com)*

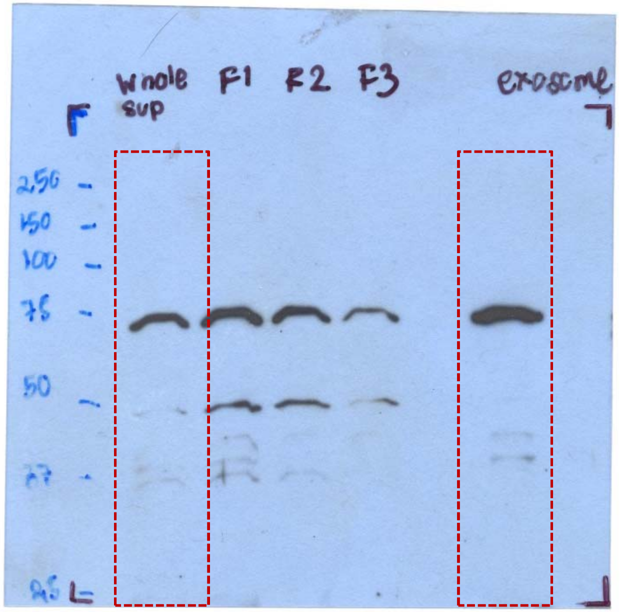

WB, HSP70, Figure 1B

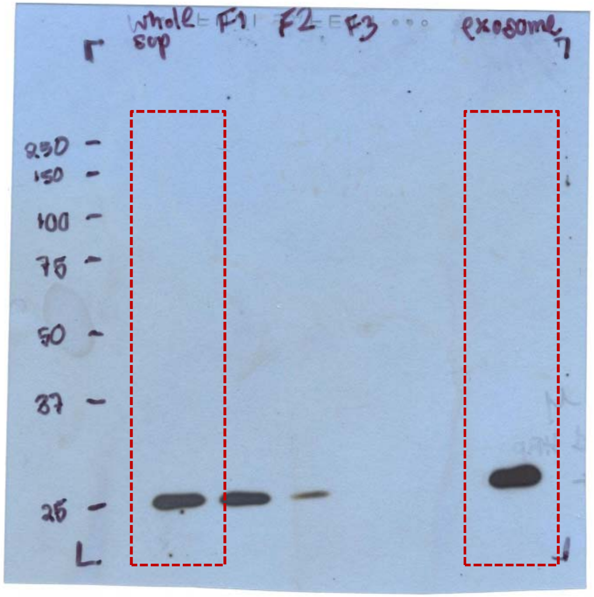

WB, Rab5, Figure 1C

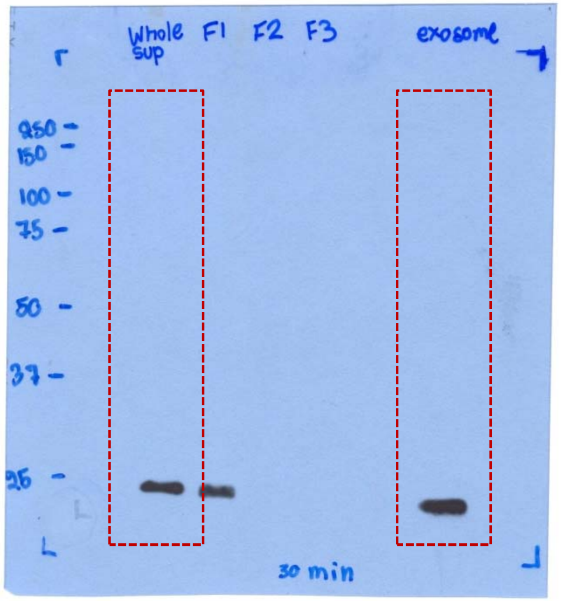

WB, Rab7, Figure 1D

Supplementary Figure S1: Original blots of the cropped images shown in **Figure 1**. The cropped areas are labeled with red-dotted boxes.
(F1, F2, F3 = excluded/washed fractions collected during exosomal isolation)
